# ID 191 - Monitoramento dos Preços de Medicamentos Incorporados no Âmbito do SUS

**DOI:** 10.5327/2237-9622.2025.v34s1.191

**Published:** 2025-11-25

**Authors:** Mariá Gonçalves Pereira da Silva, Amanda Oliveira Lyrio, Ana Carolina de Freitas Lopes, Luciene Fontes Schluckebier Bonan

**Keywords:** compras governamentais, monitoramento de preços, sustentabilidade do SUS, avaliação econômica em saúde

## Abstract

**Introdução:**

A avaliação de evidências econômicas acerca das tecnologias em saúde é determinante na sua incorporação ao Sistema Único de Saúde (SUS). A proposição do preço unitário de cada tecnologia é um parâmetro crucial na estimativa do impacto orçamentário, e o cumprimento desse preço na implementação ajuda a garantir a sustentabilidade do Sistema. Nesse sentido, esse trabalho objetiva comparar os preços propostos para incorporação com os preços praticados no primeiro ano de implementação de medicamentos no âmbito do SUS.

**Método:**

Foram incluídos os medicamentos com recomendação de incorporação ou ampliação de uso pela Comissão Nacional de Incorporação de Tecnologias no SUS (Conitec) nos anos de 2019 e 2020. Entre esses, foram considerados os que pertencem ao Componente Especializado da Assistência Farmacêutica (Ceaf), cuja compra é realizada de forma centralizada pelo Ministério da Saúde (Grupo 1A) ou compras estaduais com financiamento do Ministério da Saúde (Grupo 1B). Medicamentos que não foram implementados até agosto de 2024 ou que não possuíam informações de utilização disponíveis foram excluídos. Para a obtenção dos preços propostos, realizou-se uma busca nos Relatórios de Recomendação da Conitec. Os preços praticados foram obtidos por meio de consulta ao Banco de Preços em Saúde (BPS) e ao Sistema de Integrado de Administração e Serviços Gerais (Siasg). As buscas foram realizadas considerando compras federais do Departamento de Logística em Saúde (Dlog) e das secretarias estaduais ao longo do primeiro ano de implementação de cada medicamento, sendo excluídas as compras judiciais. A comparação entre os preços propostos e os praticados foi calculada por meio da diferença percentual.

**Resultados:**

Foram identificados 13 medicamentos do Grupo 1A e 4 do Grupo 1B. A diferença entre os preços propostos e praticados variou de -61% a 206%, com mediana de -4%. Para a maioria das tecnologias (71%, n=12), o preço praticado foi inferior ao proposto. Entre as cinco tecnologias com preço superior, para quatro delas (80%) o preço praticado era menor que o percentual de reajuste da Câmara de Regulação do Mercado de Medicamentos (Cmed) do período. O único que não foi justificado pelo ajuste da Cmed foi o omalizumabe (Xolair® – Novartis), que apresentou a maior diferença entre o preço proposto e o praticado (206%). Ressalta-se que o omalizumabe pertence ao Grupo 1B do Ceaf, logo sua compra é realizada pelas secretarias estaduais.

**Conclusão:**

Discussão e conclusões: Os resultados indicam que os preços propostos são, em geral, cumpridos ou estão abaixo do previsto no primeiro ano da implementação para maioria dos medicamentos. Dessa forma, apresenta-se como um parâmetro confiável para o cálculo de estimativas orçamentárias no processo de tomada de decisão para a incorporação de tecnologias. No entanto, o caso do omalizumabe, cujo preço foi significativamente superior ao proposto e não justificado pela Cmed, ressalta-se a necessidade de definir estratégias para evitar situações de descumprimento dos preços acordados. Além disso, sugere-se um monitoramento mais rigoroso das compras realizadas pelos estados, que, devido ao menor volume de aquisição, podem enfrentar dificuldades em negociar melhores condições de preço.

